# Research advancements on the role of CircRNAs in cartilage injury within osteoarthritis

**DOI:** 10.3389/fgene.2026.1718719

**Published:** 2026-03-06

**Authors:** Chengri Liu, Yanqun Liu, Baojian Zhang, Qingyu Xu

**Affiliations:** Orthopedic Diagnosis and Treatment Center, The Affiliated Hospital of Yanbian University (Yanbian Hospital), Yanji, Jilin, China

**Keywords:** arthritis, articularcartilageinjury, biomarkers, CircularRNAs, mechanism of action

## Abstract

The main causes of articular cartilage injury (ACI) encompass inflammation, trauma, chronic strain, degeneration, and so forth. ACI is one of the main pathological features of degenerative joint diseases such as osteoarthritis (OA), which significantly affects patients' normal work and life. Due to the absence of nerves, blood vessels, and lymphatic tissue in the cartilage, it is challenging for it to repair itself after injury.​ Non-coding RNAs, as crucial regulators of gene expression, have been increasingly implicated in the pathophysiology of various diseases. Among them, circular RNAs(circRNAs), as a new type of endogenous special non-coding RNAs, have been extensively discovered in eukaryotic cells. Owing to their unique closed-loop structure and potentially stable expression characteristics, circRNAs have demonstrated significant regulatory roles in the occurrence and development of various diseases. circRNAs are differentially expressed in OA chondrocytes and normal chondrocytes, and are involved in the inflammatory response, proliferation, apoptosis, and other processes of chondrocytes and the extracellular matrix (ECM). This article aims to review the recent research progress of circRNAs in ACI and explore their potential role in the pathogenesis of OA.

## 1 Introduction

**FIGURE 1:**
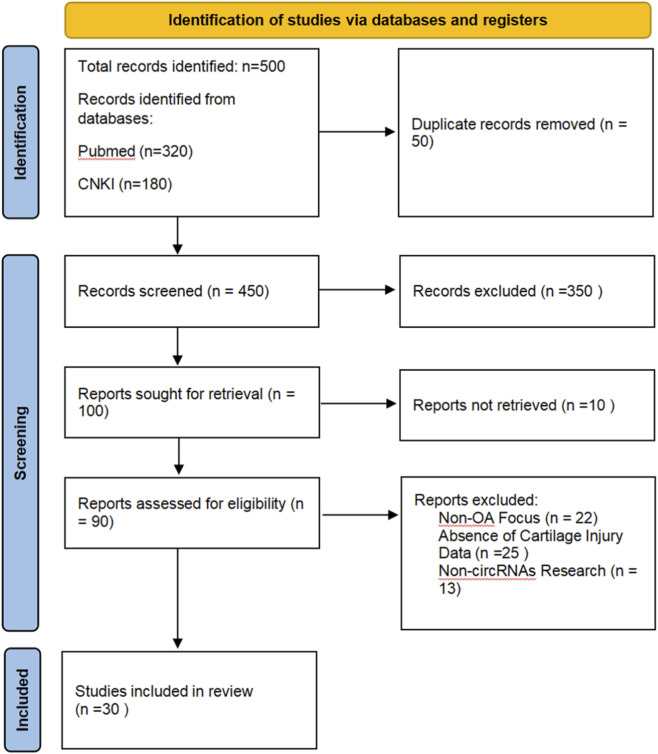
Preferred Reporting Items for Systematic Reviews and Meta-Analyses (PRISMA) flow diagram for the review selection process.

Osteoarthritis (OA) is a prevalent chronic degenerative disorder of the motor system, and the disease progresses gradually with a high rate of disability. It is typically manifested as joint swelling, stiffness, pain, joint space narrowing, and limited activity (Rayes et al., 2025). It occurs in all joints of the body, most frequently involving large load-bearing joints such as hips, knees, and ankles. It is characterized by articular cartilage degeneration, subchondral osteosclerosis, and synovial inflammatory changes (Wang et al., 2023). The destruction and degeneration of articular cartilage play a crucial role in the occurrence and development of OA. The reduction of chondrocytes and the loss of extracellular matrix (ECM) degradation lead to the lack of protection of subchondral bone, resulting in stiffness, inflammatory changes, and destruction, causing irreversible damage to the articular anatomical structure and internal environment. However, there are numerous theories regarding the pathogenesis of articular cartilage injury (ACI) in OA, but the specific pathogenesis remains unclear and requires further investigation.​ Emerging evidence highlights the pivotal roles of non-coding RNAs in regulating gene expression networks underlying OA pathogenesis. With the advancement of high-throughput sequencing technology, a large number of studies have discovered that circRNAs, as a novel class of endogenous non-coding RNAs have attracted extensive attention due to their unique closed-loop structure, which confers potentially ultra-high stability, excellent immunogenicity, and potential biological characteristics. Growing evidence indicates that circRNAs are closely associated with chondrocyte proliferation, apoptosis, inflammation, autophagy, and ECM metabolism in OA, and either delay or exacerbate the progression of OA (Mao et al., 2021). They play a significant regulatory role in the occurrence and development of articular cartilage injury and serve as biomarkers to provide guidance for disease diagnosis and treatment. This article reviews the role of circRNAs in OA cartilage injury. PRISMA is shown in Figure 1.

## 2 CircRNAs basic features

The human gene pool consists of three billion base pairs. Genome sequencing results reveal that approximately 98% of non-protein-coding genes can be transcribed into non-coding RNA (Yang et al., 2024), while only 2%–3% of the genome can be transcribed into protein-coding genes (Tian et al., 2023). CircRNAs are a special category of endogenous non-coding RNA molecules. They were initially discovered by Sanger in 1976 through electron microscopy in plant eukaryotic cells (Sanger et al., 1976), and were later identified in the mouse Sry gene in 1993 (Zhao et al., 2021). Structurally, circRNAs lack a 5′methylguanosine cap and 3′polyadenylate tail, and instead feature a covalently closed, single-stranded loop (Wei and Lu, 2019). This unique closed-loop structure is formed when pre-mRNA undergoes back-splicing catalyzed by RNA polymerase II (Pol II) (Wu et al., 2020). That is, the poly(A) tail at the 3′end of an exon of a gene is connected to the 5′end of the same exon or an adjacent exon, forming a closed ring structure through a covalent bond. This structural characteristic generally makes them unaffected by RNA exonuclease, not prone to degradation, and capable of being stably expressed in cells, although conservation across species may vary and should be verified with resources like circAtlas.

In most genes, the expression of circRNAs is 10% higher than that of mRNA, and even the expression of some circRNAs is 10 times higher than that of mRNA. Studies have indicated that the number of circRNAs in human platelets is 17–188 times that in nucleated tissues (Zhang W. J. et al., 2024), and circRNAs also demonstrate significant tissue specificity and stage specificity (He et al., 2025), which makes them an important tool for studying the expression of disease-related genes. With the advancement of high-throughput sequencing technology (Conn et al., 2024), a large number of circRNAs have been discovered. Wang H. Y, et al. (2019) identified a total of 8,321 circRNAs in the human placenta using RNA sequencing technology (RNA-SEQ). Additionally, two significant research papers published by Hansen et al. (2013); Sebastian et al. (2013) in 《Nature》 in 2013 revealed the function of circRNAs as a microRNAs (miRNAs) sponge, further promoting the upsurge in the research of circRNAs.

## 3 Classification of circRNAs

**FIGURE 2:**
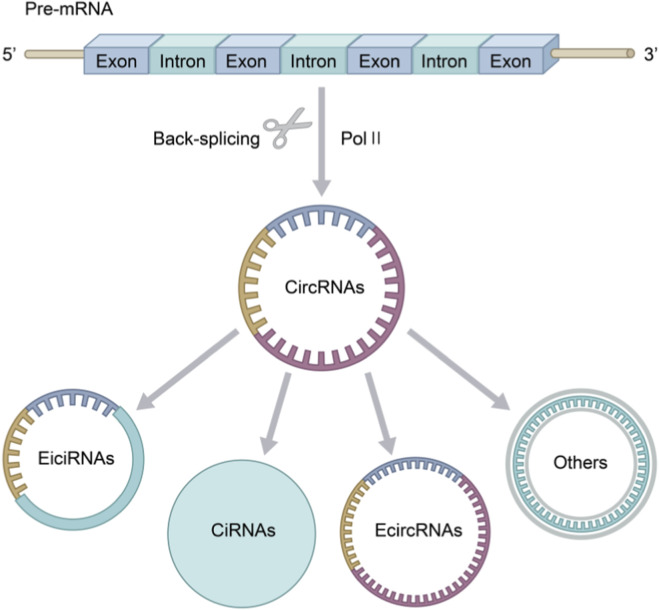
Diagram of CircRNAs formation and classification (drawn by Biorender.com).

Based on different genome and sequence composition, circRNAs can be classified into three main types:Exonic circRNAs(ecircRNAs), ntronic circRNAs(ciRNAs) and exon-in-tron circRNAs(eiciRNAs) (Yang et al., 2017). Additionally, there are some rare small subgroups, such as fusing circular RNAs (f-circRNAs), readable circular RNAs (rt-circRNAs) (Fang et al., 2021), intergenic circular RNAs and tRNA intron circRNAs (Chen et al., 2022). Among these circRNAs, ecircRNAs account for approximately 80%, mainly existing in the cytoplasm and playing a significant role in the occurrence and development of ACI shown in Figure 2 (Wang et al., 2020).

## 4 Biological characteristics of CircRNAs

**FIGURE 3:**
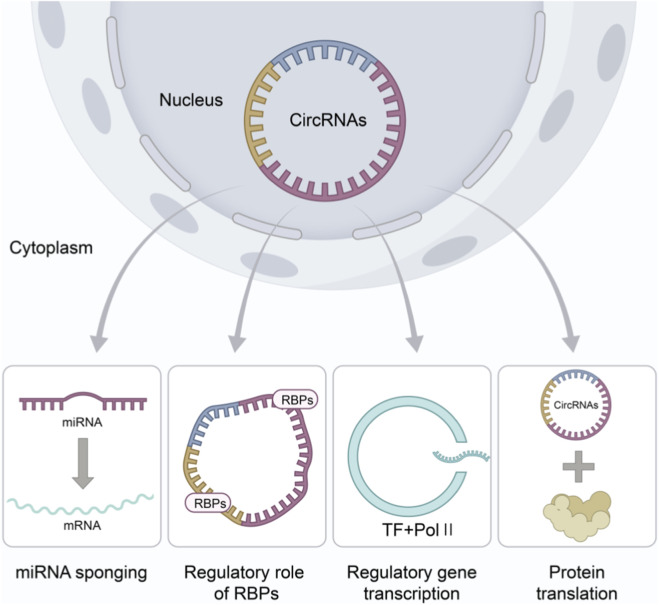
Biological function diagram of CircRNAs (drawn by Biorender.com).

CircRNAs are single-stranded, covalently closed RNA structures formed through the backsplicing of precursor mRNAs. They exert their biological functions primarily via four molecular mechanisms (Figure 3): (1) Acting as “molecular sponges” for miRNAs, they sequester miRNAs and thereby alleviate the repression of target mRNAs (Ni et al., 2019; Hu et al., 2022). (2) They interact with RNA-binding proteins (RBPs) to form RNA-protein complexes (RPCs) (Chen et al., 2019; Cheng and Han, 2022), influencing various cellular processes. (3) Certain circRNAs contain internal ribosome entry sites (IRESs) that enable cap-independent translation, leading to the production of functional peptides (Fernandes et al., 2025; Wang et al., 2025). (4) Nuclear-localized circRNAs (such as eiciRNAs) can interact with RNA polymerase II to regulate the transcription of their parent genes (Sanadgol et al., 2024; Wan et al., 2024). These characteristics establish circRNAs as important players in the regulation of gene expression.These functional mechanisms are not isolated; extensive cross talk exists among them, enabling circRNAs to form complex regulatory networks. For instance, a single circRNA can simultaneously function as a miRNA sponge and bind to RBPs, thereby integrating miRNA-mediated post-transcriptional control with RBP-mediated processes such as mRNA stability, splicing, or localization. Moreover, the peptides translated from certain circRNAs may themselves interact with or modulate the activity of proteins involved in the same or related pathways, creating feedback or feed-forward loops.

## 5 The role of CircRNAs in ACI

### 5.1 Regulation of ECM synthesis and decomposition

The extracellular matrix (ECM) plays a crucial role in supporting the structure of chondrocytes and, as a medium for transmitting growth factors and chondrodevelopmental signaling molecules, regulates the growth and development of chondrocytes (Jin and Luo, 2019). ECM is mainly composed of Aggrecan (AGC) and collagen II (Col II) (Wang et al., 2018). The imbalance between catabolism and anabolism in ECM will result in articular cartilage degeneration, which is also one of the main mechanisms of OA. circRNAs can act as miRNA sponges to competitively inhibit the function of miRNAs (Umer et al., 2025; Guo et al., 2025). For instance, circPDE4D serves as a sponge to eliminate the function of miR-103a-3p through direct binding and induce ECM degradation. Disintegrins and metalloproteinases (ADAMTS) and matrix metalloproteinases (MMPs) with thromboreactive protein-like motifs are accountable for ECM degradation (Wang et al., 2021). MMPs, such as MMP-13 (which mainly degrades Col II) and MMP-9, are key proteases accountable for ECM degradation. Circular RNA CREBBP has been demonstrated to activate the Smad1/5 pathway through the TGF-β2/ALK1 axis, and the competitive endogenous RNA network formed by circCREBBP acts as a miR-1208 sponge, promoting the expression of TGF-β2 by phosphorylation of Smad1/5. This exacerbates ECM degradation and cartilage degeneration both *in vivo* and *in vitro*, thereby facilitating the progression of OA (Xu et al., 2022). Some other studies have also confirmed this change (Wang et al., 2024). The knockdown of CCN2-derived circRNAs significantly reduces the synthesis of aggregator mRNA and proteoglycan, thereby diminishing the synthesis capacity of ECM (Soma et al., 2023). circRNA-MSR and circRNA-CER are highly expressed in the area of damaged cartilage in OA. After the knockdown of circRNA-MSR and circRNA-CER, the expression of TNF-α is inhibited, and the contents of Col II and AGC are increased, indicating that circRNA-MSR and circRNA-CER can promote the degradation of ECM (Wang et al., 2021; Jia et al., 2022). The damage of the joint is aggravated and the protective effect of subchondral bone is lost.

Fibroblast growth factor 18(FGF18), which is the target of circPDE4D and miR-103a-3p, is capable of enhancing anabolism, inhibiting catabolism, preventing and reducing ECM degradation, and promoting ECM formation (Mao et al., 2021). Jiarui et al. (2023) demonstrated that circARPC1B is downregulated in cholesterol-treated chondrocytes and plays a crucial role in the preservation of ECM. From a mechanical perspective, circARPC1B competitively binds to the E3 ligase SYVN1 binding site on vimentin, suppresses the proteasomal degradation of vimentin, and protects joint soft cells and ECM. Zhou Z. B. et al. (2020) employed related adenoviruses to reveal that the upregulated expression of circCDH13 in the medial meniscus (DMM)-induced OA cartilage tissue of mice can significantly induce chondrocyte apoptosis, promote ECM catabolism, and inhibit ECM anabolism. Silencing circCDH13 *in vivo* significantly alleviates DPM-induced OA in mice, reduces chondrocyte apoptosis, inhibits ECM catabolism, and promotes ECM anabolism through the miR-296-3p-PTEN pathway. Experiments have also indicated that carnosic acid can significantly improve IL-1β-induced chondrocyte matrix degradation and inhibit the nuclear factor-κB (NF-κB) signaling pathway through P65 (Huang and Zhou, 2024). By regulating the various pathways and processes of ECM in articular cartilage via circRNAs, the expression of ECM can be upregulated or downregulated, and the release of corresponding degrading proteases can be inhibited, thereby achieving the inhibition of ECM catabolism, enhancement of anabolism, and increase in the content of proteoglycan and collagen. This strengthens the scaffold structure of chondrocytes, enhances the wear resistance of cartilage in the joint, and reduces the degree of injury.

### 5.2 Regulation of chondrocyte proliferation, differentiation and apoptosis

Apoptosis is one of the crucial mechanisms implicated in ACI and leading to OA. With the continuous enhancement of detection methods and levels, the observation of chondrocyte apoptosis has become more meticulous. Flow cytometry, 3-(4, 5-dimethylthiazole-2-yl)-5-(3-carboxymethoxyphenyl)-2-(4-sulfophenyl)-2H-tetrazole, five-ethyl-2′-deoxyuridine, and Annexin V-fluorescein isothiocyanate apoptosis assay can be utilized to assess apoptosis (Gao et al., 2022; Zhang S. X. et al., 2022). Numerous studies have indicated that circRNAs plays a significant role in regulating the proliferation, differentiation, and apoptosis of chondrocytes. Zhang J. J. et al. (2024) discovered that silencing circ_0136474 could promote cell proliferation and cell cycle progression, prolong the S phase, inhibit cell apoptosis, and reduce the release of IL-6, TNF-α, and MMPs, suggesting that knocking out the circ_0136474 gene could protect chondrocytes from injury. Shen et al. (2020) found in the rabbit model that circCDK14 downregulated the expression of Smad2 through the adsorption of miR-125a-5p “sponge”, resulting in the dysfunction of the TGF-β signaling pathway. Thus, the downregulation of circCDK14 in the ACI position simultaneously regulated the metabolism of cartilage and inhibited cell apoptosis. It can promote the differentiation and proliferation of chondrocytes and effectively alleviate OA. Gou et al. (2023) demonstrated through rat experiments that circ_0038467 could promote the maturation of miR-203, increase the apoptosis of chondrocytes induced by lipopolysaccharide, and accelerate the progression of OA. However, by knocking out the circ_0038467 gene or inhibiting its overexpression, the occurrence and development of ACI can be effectively controlled, and OA symptoms can be mitigated and improved. Similarly, Li et al. (2022) in the human rat model study also confirmed that circRNA VMA21 could improve IL-1β-induced chondrocyte injury through the miR-495-3p/FBWX7 signaling axis, and the strong expression of circRNA VMA21 enhanced chondrocyte viability. The apoptotic process was attenuated, along with the upregulation of Bcl-2 and the downregulation of Bax and C-caspase-3. In addition to animal experiments to verify the regulatory effect of circRNAs, *in vitro* experimental studies on human OA cartilage also confirmed that the expression of hsa_circ_0023404 in OA chondrocytes was abnormally elevated. Down-regulating the expression of hsa_circ_0023404 can promote the proliferation and inhibit the apoptosis of human OA chondrocytes, which may be related to inhibiting the secretion of inflammatory factors and the JAK/STAT signaling pathway (Li et al., 2016). According to *in vitro* experiments on human knee cartilage, circ-BRWD1 targets and negatively regulates miR-488-3p, and the inhibition of miR-488-3p can reverse the effects of circ-BRWD1 knockdown on the proliferation and apoptosis of OA chondrocytes. It is suggested that circ-BRWD1 is involved in the development of knee osteoarthritis by targeting and negatively regulating miR-488-3p (Shen et al., 2019). In the circRNAs epigenetic gene chip screening of hADSC chondrogenic differentiation glomerulus samples, it was revealed that the expression level of cir-cAZIN1 was significantly upregulated, which had the effect of inhibiting chondrocyte degeneration. It was disclosed that cir-cAZIN1 inhibited the silencing effect of CACNA1I through sponge adsorption of hsa-miR-654-3p and thus played a role in inhibiting chondrocyte degeneration (Wang X. J. et al., 2019).

In contrast, the downregulation of circANKRD36 under IL-1β stress can enhance the apoptosis and inflammation of chondrocytes (Zhou J. L. et al., 2020). circ_0136474 and MMP-13 inhibit cell proliferation and promote apoptosis by competing to bind to miR-127-5p (Ou et al., 2019). circ_0040646 regulates the proliferation, apoptosis, and differentiation of knee osteoarthritis chondrocytes by targeting miR-188-3p inhibition. The low expression of circ_0040646 can facilitate the proliferation of knee osteoarthritis chondrocytes, while its overexpression can suppress the proliferation of such chondrocytes. circ_0040646 may serve as an intervention target for the treatment of knee osteoarthritis (Zhang X. P. et al., 2022). By up-regulating or silencing the regulation of circRNAs on chondrocytes, the cell cycle process is influenced, cell proliferation and differentiation are promoted, and apoptosis is inhibited, which can effectively control and alleviate the development of OA caused by ACI. At the same time, it can reverse the disease to a certain extent and achieve the purpose of repairing the damage and treating bone and joint diseases.

### 5.3 The role of CircRNAs in inflammation

OA induced by TCI is associated with inflammation, involving a variety of pro-inflammatory mediators, such as IL-1β, TNF-α, NF-κB, and other chemokines, like nicotinamide phosphoribosyl transferase. Stimulated by the inflammatory factor IL-1β, I-κBα in the NF-κB signaling pathway is phosphorylated and degraded, leading to the translocation of its bound P65 from the cytoplasm to the nucleus and promoting the expression of inflammatory mediators and catabolic enzymes (Zheng et al., 2023). Under the influence of inflammation, chondrocytes lose the scaffold function of ECM, and a large number of cell cycles are arrested, with necrosis and apoptosis occurring. IL-1β and TNF-α were also discovered to be classical pro-inflammatory factors in animal arthritis models, and their signaling pathways were mostly strom-degrading enzymes or cell cycle effector proteins (Xu et al., 2021). Under the stimulation of inflammatory cytokines IL-1β and TNF-α, the secretion of MMPs(including MMP-13, MMP-9, etc.) and proteoglanase in chondrocytes increases, facilitating the occurrence and progression of OA through catabolism. Pro-inflammatory cytokines can enhance the expression of circIRAK3, and the increase of circIRAK3, in turn, can promote the degradation of pro-inflammatory cytokine mRNA by binding to HNRNP-U, thereby inhibiting the inflammatory response and reducing the damage to chondrocytes (Wen et al., 2023). Liang et al. (2023) studied the interaction between circ_0114876 and its downstream target miR-1227-3p and confirmed that circ_0114876 promoted the proliferation of chondrocytes and inhibited apoptosis and inflammation. CircSLTM and high mobility histone B2 (HMGB2) are upregulated in IL-1β-induced chondrocytes, while Mir-421 is downregulated. Knocking down circSLTM or overexpressing miR-421 can improve IL-1β-induced chondrocyte apoptosis and inflammatory response (Zhang et al., 2023). Li et al. (2025) research show that environmentally responsive drug delivery system based on circ-srIκBα shows significant potential for OA treatment, offering improved cell specificity, stability, and low immunogenicity. Some circRNAs, such as CircRNA_09505, are upregulated CircRNAs that promote the expression of AKT1 in macrophages through the miR-6089/IκBα/NFκB signaling pathway, thereby exacerbating inflammation and joint injury (Chen et al., 2021). Bu et al. (2025) findings indicate that circPAFAH1B2 acts as a molecular decoy blocking ClpB mitochondrial translocation, driving mitochondria-dependent cartilage degradation.As endogenous competing RNA, circRNAs absorb corresponding miRNAs through the “sponge” effect, relieve the inhibition of miRNAs on their target mRNA, regulate mRNA expression, and indirectly regulate the expression level of the downstream target gene insulin-like growth factor 1 (IGF-1) receptor, and participate in the pathogenesis of OA (Liu et al., 2019).

### 5.4 The role of CircRNAs autophagy

**FIGURE 4:**
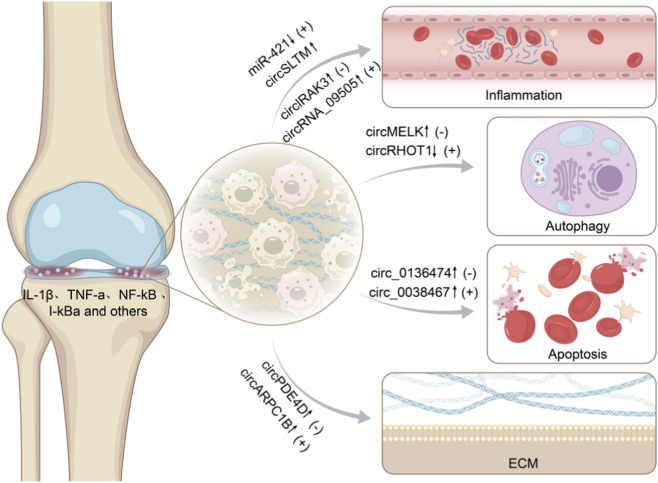
The role of CircRNAs in ACI (drawn by Biorender.com).

Autophagy is a common Programmed Cell Death (PCD) process. Through phagocytosis, harmful or useless components such as abnormal cytoplasmic proteins or organelles are gathered and packaged into vesicles, which fuse with lysosomes to form autophagolysosomes and degrade their contents. This is done to meet the metabolic requirements of the cell itself and the renewal of certain organelles, ensuring the survival and functionality of normal cells, enabling cells to adapt to changes in the internal and external environment. Autophagy represents the capacity of cells to prevent their own demise and safeguard cells from apoptosis. The autophagy process relies on the formation of various complexes on the membrane and can be divided into initiation, nucleation, elongation, autophagosome-lysosome fusion, and degradation. Intracellular signaling pathways that regulate autophagy can either inhibit or activate it. AMPK, PI3K/AKT, mTOR, MAPK/ERK, and BCL2 regulate autophagy by influencing the expression of key proteins like the ULK1 complex, the PI3K complex, the ATG protein family, and LC3-II (Zhang et al., 2019). circRNAs can alleviate and reverse abnormal autophagy behavior and exert a protective effect (Tang et al., 2023). circMELK, as the “sponge” of miR-497-5p, regulates the MYD88/NF-κB signaling axis, promotes pathway activation, and subsequently promotes OA chondrocyte apoptosis and inhibits autophagy (Zhang Y. C. et al., 2022). In OA rats, the loss of circRHOT1 enhanced the expression of Col II and aggregin, inhibited cell viability and proliferation, but induced apoptosis of chondrocytes. Low circRHOT1 can induce the expression levels of LC3, beclin1, Col II, and aggregin (Man et al., 2022). It enhances the autophagy renewal capacity of chondrocytes, promotes metabolism, and creates a favorable internal environment for the repair of arthritis ACI. Through the regulatory role of circRNAs in autophagy, damaged, infected, and senescent cells are eliminated to maintain normal human development and the stability of the internal environment (Gao et al., 2024). Autophagy is an important PCD pathway. Under the regulation of various autophagy-related genes, autophagosomes are formed by lysosomes to degrade cellular contents. circRNAs can regulate the biological activity of chondrocytes by influencing the autophagy pathway, and play a dual role in either inhibiting or promoting the occurrence and development of diseases shown in Figure 4.

## 6 Summary and prospect

OA is one of the most prevalent chronic bone and joint disorders, imposing substantial impacts on patients' quality of life and socioeconomic burden. ACI, characterized by chondrocyte destruction, apoptosis, and ECM degradation, represents a core pathological feature of OA. Current therapeutic strategies primarily focus on repairing ACI to delay disease progression. With advances in high-throughput sequencing, numerous circRNAs have been identified as endogenous non-coding RNAs formed via back-splicing, showing differential expression in OA. These molecules function through miRNA sponging, pathway regulation, and protein interactions, influencing chondrocyte proliferation, apoptosis, inflammation, and autophagy. Although growing evidence underscores the involvement of circRNAs in OA pathogenesis, several critical aspects require deeper analysis and translational consideration.

### 6.1 Consensus and controversies in CircRNAs mechanisms

Table 1 summarizes key circRNAs implicated in ACI, yet their roles are often context-dependent and occasionally paradoxical. For instance, while circCDH13 silencing alleviates OA by inhibiting apoptosis and ECM catabolism via the miR-296-3p/PTEN axis, circ_0038467 promotes apoptosis through miR-203 sponging. Such opposing effects highlight the complexity of circRNA-mediated regulation and potential pathway crosstalk (e.g., TGF-β/Smad, NF-κB). Notably, circRNAs like circIRAK3 exhibit anti-inflammatory properties, whereas circRNA_09505 exacerbates inflammation via the miR-6089/IkBα/NF-κB pathway. These contradictions underscore the need to elucidate tissue-specific modulators and upstream regulators of circRNA expression. Future studies should integrate multi-omics data to resolve these discrepancies and map interactive networks governing OA progression.

**TABLE 1:** Expression and signaling pathway of CircRNAs in ACI.

CircRNAs	Express	Mechanism	Function	References
circPDE4D	Up	miR-103a-3p	ECM degradation	Wang et al. (2021)
circRNA CREBBP	Up	miR-1208	ECM degradation and cartilage degeneration	Xu et al. (2022)
CCN2-derived circRNA	Up	miR-181-5p	Aggregated mRNA and proteoglycan synthesis	Soma et al. (2023)
circRNA-MSR	Up	miR-761	Inhibit the autophagy of OA chondrocytes and promote the degradation of ECM	Wang et al. (2021), Jia et al. (2022)
circRNA-CER	Up	miR-136	ECM degradation	Wang et al. (2021)
CircARPC1B	Down	Cholesterol-circARPC1B-Vimentin axis	Protect joint soft cells and ECM	Jiarui et al. (2023)
CircCDH13	Down	miR-296-3p-PTEN	Inhibit chondrocyte apoptosis, ECM decomposition, promote ECM synthesis	Zhou et al. (2020a)
circ_0136474	Up	miR-127-5p	Inhibit chondrocyte proliferation and stimulate cell apoptosis	Zhang et al. (2024b)
circCDK14	Down	miR-125a-5p	Inhibit apoptosis and promoted the differentiation and proliferation of chondrocytes	Shen et al. (2020)
circ_0038467	Up	miR-203	Augment the apoptosis of chondrocytes induced by lipopolysaccharide and advance the progression of OA.	Gou et al. (2023)
Circular RNA VMA21	Up	miR-495-3p/FBWX7	Enhance the activity of chondrocytes and mitigate apoptosis	Li et al. (2022)
hsa_circ_0023404	Up	JAK/STAT signal pathway is inhibited	Inhibit chondrocyte proliferation and boost cell apoptosis	Li et al. (2016)
circ-BRWD1	Down	miR-488-3p	Inhibit the proliferation of chondrocytes and expedite the development of arthritis	Shen et al. (2019)
cir-cAZIN1	Up	hsa-miR-654-3p	Inhibit chondrocyte degeneration	Wang et al. (2019b)
circANKRD36	Down	miR-599	Promote chondrocyte apoptosis and inflammation	Zhou et al. (2020b)
circ_0136474	Up	miR-127-5p	Inhibit cell proliferation and induce cell apoptosis	Ou et al. (2019)
circ_0040646	Down	miR-188-3p	Promote chondrocyte proliferation	Zhang et al. (2022b)
circIRAK3	Up	HNRNP U	Promote the degradation of pro-inflammatory cytokine mRNA, thereby suppressing the inflammatory response and reducing the damage to chondrocytes	Wen et al. (2023)
circ_0114876	Up	miR-1227-3p	Promote chondrocyte proliferation, inhibit cell apoptosis and inflammatory reaction	Liang et al. (2023)
CircSLTM	Up	miR‐421	It exacerbates the apoptosis and inflammatory reaction of chondrocytes	Zhang et al. (2023)
CircRNA_09505	Up	miR-6089	Intensify inflammation and joint damage	Chen et al. (2021)
circMELK	Up	miR-497-5p	Promote OA chondrocyte apoptosis and inhibit autophagy	Zhang et al. (2022c)
circRHOT1	Up	miR-142-5p	Inhibit cell viability and proliferation and induce apoptosis of chondrocytes	Man et al. (2022)

Furthermore, the interaction network among non-coding RNAs, particularly the crosstalk between circular RNAs (circRNAs), Piwi-interacting RNAs (piRNAs), and microRNAs (miRNAs), forms a complex regulatory basis for modulating downstream target genes (Zhuang and June 2023; Kong et al., 2021). For instance, certain circRNAs can function as competitive endogenous RNAs (ceRNAs) that sequester specific miRNAs via a “molecular sponge” mechanism, thereby relieving their repression on target mRNAs and influencing critical pathological processes such as chondrocyte proliferation, apoptosis, inflammation, and extracellular matrix metabolism (Zhuang and June 2023). This ceRNA regulatory network does not operate in isolation and may interact with other non-coding RNA systems, such as piRNAs. Research has indicated that in osteoarthritis, circRNAs, long non-coding RNAs (lncRNAs), and miRNAs form an extensive interaction network that fine-tunes the expression of key genes, including SOX9, MMP13, and ADAMTS5, thereby profoundly influencing disease progression (Kong et al., 2021). Understanding how these non-coding RNA components coordinate or antagonize each other is crucial for elucidating the complete molecular mechanisms of osteoarthritis and identifying novel therapeutic targets. Future studies should further integrate multi-omics data to decipher these intricate regulatory circuits and clarify the dominant roles of different non-coding RNAs in specific cell types and disease stages.

### 6.2 CircRNAs as biomarkers and clinical translation challenges

CircRNAs hold promise as OA biomarkers due to their potential stability and detectability in bodily fluids, though conservation and specificity should be assessed using tools like circAtlas for clinical application.However, clinical application faces hurdles.In terms of specificity and sensitivity, while circRNAs like hsa_circ_0023404 show differential expression in OA chondrocytes, their diagnostic accuracy (e.g., AUC values) in human synovial fluid or serum remains underexplored. Studies correlating circRNAs levels with radiographic OA stages (e.g., Kellgren-Lawrence grading) are scarce but essential. For instance, future work should validate circRNA signatures against Kellgren-Lawrence grades to establish stage-specific biomarkers.​ For sample stability, circRNAs integrity in synovial fluid under varying storage conditions and disease phases requires standardization. On technical barriers, current detection methods (e.g., RNA-seq, qPCR) need enhanced sensitivity to distinguish low-abundance circRNAs from linear isoforms in clinical samples. Emerging technologies like spatial transcriptomics could map circRNAs distribution across cartilage zones, providing insights into zone-specific roles in OA.For example, recent spatial transcriptomics studies have revealed distinct circRNA expression patterns in superficial versus deep cartilage zones in OA, highlighting their potential as zone-specific regulators.

### 6.3 Barriers to therapeutic application

Many circRNAs exhibit tissue-restricted expression, which may confound systemic biomarker utility or off-target effects in gene therapies. Viral vectors (e.g., AAVs) for circRNAs mimic/inhibitor delivery pose immunogenicity risks, while non-viral carriers (e.g., nanoparticles, hydrogels) require optimization for cartilage penetration and cellular uptake. CircRNAs sponges may inadvertently regulate non-target miRNAs or genes, necessitating CRISPR-based precision editing or chemically modified oligonucleotides. Overcoming these barriers will require collaborative efforts in biomaterial engineering and preclinical models to assess long-term safety and efficacy.

### 6.4 Future directions

Prospects for circRNA-based OA interventions, utilizing single-cell circRNAs sequencing and spatial transcriptomics to resolve heterogeneity in chondrocyte subpopulations and OA stages. Spatial transcriptomics has enabled mapping of circRNAs like circPDE4D in OA cartilage zones, correlating with degradation gradients. Linking circRNAs dynamics to ongoing clinical trials (e.g., RNA-based therapies) and patient outcomes. Designing circRNA-overexpressing vectors or knockdown strategies coupled with targeted delivery systems to enhance specificity and minimize immune responses.In conclusion, while circRNAs offer novel insights into OA pathogenesis and potential diagnostic/therapeutic avenues, addressing mechanistic controversies, clinical validation gaps, and delivery challenges is crucial. Future research should prioritize translational studies to harness circRNAs for safe and effective OA management.
